# Sample size estimation for task-related functional MRI studies using Bayesian updating^☆^

**DOI:** 10.1016/j.dcn.2024.101489

**Published:** 2024-12-17

**Authors:** Eduard T. Klapwijk, Joran Jongerling, Herbert Hoijtink, Eveline A. Crone

**Affiliations:** a Erasmus University Rotterdam, Netherlands; b Tilburg University, Netherlands; c Utrecht University, Netherlands; d Leiden University, Netherlands

**Keywords:** Power analysis, Region of interest, Effect size, R package, Sample sizes, Bayesian updating

## Abstract

Task-related functional MRI (fMRI) studies need to be properly powered with an adequate sample size to reliably detect effects of interest. But for most fMRI studies, it is not straightforward to determine a proper sample size using power calculations based on published effect sizes. Here, we present an alternative approach of sample size estimation with empirical Bayesian updating. First, this method provides an estimate of the required sample size using existing data from a similar task and similar region of interest. Using this estimate researchers can plan their research project, and report empirically determined sample size estimations in their research proposal or pre-registration. Second, researchers can expand the sample size estimations with new data. We illustrate this approach using four existing fMRI data sets where Cohen’s d is the effect size of interest for the hemodynamic response in the task condition of interest versus a control condition, and where a Pearson correlation between task effect and age is the covariate of interest. We show that sample sizes to reliably detect effects differ between various tasks and regions of interest. We provide an R package to allow researchers to use Bayesian updating with other task-related fMRI studies.

## 1 Introduction

Since its emergence, functional magnetic resonance imaging (fMRI) has provided unprecedented opportunities to study functional brain development during childhood and adolescence by scanning children from the age of four years onward. There is great progression in the assessment of neural functional growth using cross-sectional and longitudinal assessments of cognitive, social and affective processes across the full range of childhood to adulthood. Despite the advancements in the ability to study the developing brain in vivo using fMRI, recent years have seen an increased concern about the replicability of scientific findings in general and particularly in psychology and cognitive neuroscience (Bishop, 2019; Klapwijk et al., 2021; Munafò et al. 2017; Poldrack et al., 2017; see Marek et al. (2022) for a similar concern for resting-state functional connectivity and structural MRI data sets).

Low statistical power due to small sample sizes is arguably one of the main reasons for lower than desired replicability of study results. Statistical power is the probability that a study will reject the null hypothesis when it is false, that is, when there is a non-zero effect (e.g., Cohen’s d or Pearson’s correlation) in the population of interest. Power is determined by the size of the effect, the alpha level chosen, and the size of the sample (Cohen, 1992). The smaller each of effect size, alpha level, and sample size are, the lower the power. Since many psychological phenomena consist of relatively subtle, small effects (Funder and Ozer, 2019, Gignac and Szodorai, 2016, Yarkoni, 2009), the main source for increasing power, and over which researchers have a reasonable degree of control, is the sample size (given limited flexibility of the alpha level). Despite the need for well-powered studies to reliably detect effects of interest, empirical reports have shown that most cognitive neuroscience and fMRI studies are underpowered due to small sample sizes (Button et al., 2013, Maxwell, 2004, Nord et al., 2017, Poldrack et al., 2017, Szucs and Ioannidis, 2017, Turner et al., 2018). With developmental populations, sufficiently large sample sizes might be even harder to establish because of the challenges in recruitment and testing of young participants (Achterberg, van der Meulen, 2019; Klapwijk et al., 2021).

Recently, well-coordinated efforts and funding have led to several large-scale projects that collect developmental task-related fMRI data with larger sample sizes. These projects have the potential to resolve the problem of power with sample sizes in the thousands, such as the IMAGEN study (N≈2,000) (Schumann et al. 2010), the Philadelphia Neurodevelopmental Cohort (N≈1,000) (Satterthwaite et al. 2016), the Human Connectome Project in Development (HCP-D; N≈1,300) (Somerville et al. 2018), and the Adolescent Brain Cognitive Development (ABCD) study (N≈11,000) (Casey et al. 2018). The leap forward made with this wealth of high-powered, mostly publicly available data can hardly be overstated, given that they provide an important open research tool for researchers across the globe.

However, most fMRI studies are carried out by individual research groups in much smaller samples. These labs have the important advantage over large scale cohort studies of having opportunities to pursue new scientific questions, for example using novel paradigms. It is also vital that the findings of such studies are replicable and meaningful, meaning that these studies should be properly powered, also without sample sizes in the range of large multi-lab studies. The main issue with power analysis is that the effect size in the population of interest is unknown. One option is to use effect sizes reported in the literature of the research area at hand. But these effect sizes are often inflated due to publication bias (Gelman and Carlin, 2014, Ioannidis, 2005, Open Science Collaboration, 2015, Wicherts et al., 2016, Yarkoni, 2009). Therefore, calculating power based on published effect sizes usually underestimates the sample size needed to reliably detect an effect.

This paper will present a novel method to determine the required sample size for fMRI studies based on existing (i.e., already collected) data using Bayesian updating (Rouder, 2014). For novel paradigms with no existing data available, the method can also be used to monitor sample size requirements during data collection. Specifically, the approach will determine the proportion of the already collected data that is needed to get a desired credible interval (the Bayesian counterpart of the confidence interval). This will provide an estimate of the percentage of cases for which the credible interval is expected to be in the desired range (e.g., the interval should *not* contain the value 0 for the parameter of interest). This in turn gives us an estimate of the sample size needed for a certain level of power. The current paper will provide examples (including an R package) for two effect sizes: Cohen’s d and Pearson’s correlation. The sample size determined using existing data is valuable when designing a new research project and when justifying sample sizes in a pre-registration or in proposals sent to a (medical) ethical committee.

We will illustrate sample size determination using existing data sets and tasks that are currently widely used in the developmental fMRI literature, specifically cognitive control, reward activity, and social-cognitive processing (see Table 1 for an overview) based on existing data from our own lab. It will be determined how large the sample size should be to detect a non-zero Cohen’s d value, such that 95 % does not contain the value zero for a specific condition effect (e.g., brain activity during feedback processing versus rule application). Most prior developmental fMRI studies addressed the question whether an effect linearly increases or decreases with age (Crone and Steinbeis, 2017). We therefore also determine the sample size needed to detect a Pearson correlation of an effect with linear age that is larger than zero. In the next sections, we will first introduce Bayesian updating, the highest density credible interval, and sample size determination. Next, we will provide examples using existing data from four fMRI studies (Braams et al., 2014, Peters and Crone, 2017, Spaans et al., 2023, Cruijsen et al., 2023), and illustrate sample size determination using these examples.

**Table 1:** Overview of tasks processed in the current study.

task	source	associated papers	N	age range	cognitive process	region of interest
Feedback	BrainTime study, Leiden University	(Peters and Crone, 2017, Crone et al., 2016)	271	8–25 yrs	feedback learning	DLPFC
Gambling	BrainTime study, Leiden University	(Braams et al., 2014, Crone et al., 2016)	221	12–28 yrs	reward-processing	NAcc
Self-evaluations	Self-Concept study, Leiden University	(Cruijsen et al., 2023, Crone et al., 2022)	149	11–21 yrs	self processing	mPFC
Vicarious charity	Self-Concept study, Leiden University	(Spaans et al., 2023, Crone et al., 2022)	156	11–21 yrs	vicarious rewards	NAcc

### 1.1 Bayesian updating

Bayesian updating can be used to determine the sample size required to estimate Cohen’s d or Pearson’s correlation with a certain precision. Precision is presented in the form of a 95 % highest density credible interval (HDCI), that is, the narrowest possible interval that is believed to contain the true value of the parameter of interest with a probability of 0.95.^1^ The narrower this interval, the higher the precision with which the parameter is estimated.

Bayesian updating as implemented here relies on the assumption that a priori, that is, before collecting the data, each possible value of the parameter of interest is equally likely. This has two implications. First, the HDCI is completely determined by the data and not by prior information. Secondly, the numerical values of the estimate and endpoints of the HDCI are equal to the estimate and endpoints of the classical confidence interval.

Bayesian updating can be used to determine the smallest sample size for which the resulting HDCI does not contain the value zero. Bayesian updating consists of four steps:

1. Determine the maximum achievable size of the sample.
2. Collect the data for an initial number of participants and then compute the estimate and HDCI. The actual number chosen is irrelevant, but with 20 participants the estimate and HDCI will usually give a first impression of the size of Cohen’s d or Pearson’s correlation that is not totally determined by sample fluctuations and/or outliers.
3. Add several participants to the sample (updating) and recompute the estimate and HDCI.
4. If the HDCI still contains the value zero and the maximum achievable sample size has not been obtained, return to the previous step. Otherwise, the updating is finished and estimates and corresponding HDCI’s are reported.

#### 1.1.1 The highest density credible interval (HDCI)

It is important to highlight that the HDCI is not a confidence interval. If many data sets are sampled from the population of interest and each data set is used to compute the 95 % confidence interval for the parameter of interest, then it holds that 95 % of the intervals contain the true value. However, in contrast to HDCI’s, confidence intervals cannot be updated because their coverage level will become smaller than 95 %. This will be illustrated using a simple example.

Imagine many researchers want to show that Cohen’s d is unequal to zero. Each of them samples a data set with n=20 for each group from the population of interest (in which Cohen’s d happens to equal zero). About 5 % of the 95 % confidence intervals do not contain the value 0. These researchers will not continue their efforts; they have “shown” that “their” effect is not zero. At this stage the Type I error rate is.05. However, the 95 % remaining researchers increase their power by updating their data with another 10 persons per group and recompute their 95 % confidence intervals of which about 2.8 % (number determined using simulation) does not contain the value 0. Therefore, the Type I error rate is increased to 5 % + 2.8 % = 7.8 %, that is, the updating rendered 92.2 % confidence intervals and not 95 % confidence intervals.

A HDCI should therefore not be interpreted in the context of many hypothetical data sets sampled from the population of interest. The HDCI is computed using the observed data at hand and is the shortest interval that is believed to contain the true value of Cohen’s d with a probability of.95. When updating, the size of the data at hand becomes larger, the information with respect to Cohen’s d increases, and therefore the width of the HDCI becomes smaller. The HDCI summarizes the evidence in the data at hand with respect to the size of Cohen’s d, which is different from a confidence interval which aims to control error rates under (hypothetical) repeated sampling from the same population.

### 1.2 Sample size determination

The procedure elaborated in this paper is Bayesian (because HDCI’s are used) empirical (because existing data are used) sample size determination. We use an existing dataset of a certain size, and for all sample sizes between a minimum (e.g., n=20) and maximum sample size of the sample at hand we compute the HDCI. To account for the arbitrary order of the participant in a data set, this is done for a set number of permutations (e.g., 1000) of the participants in the data set. For each sample size, we then calculate the average HDCI of all permutations. Considering both the average HDCI and the HDCI’s resulting from the different permutations, will provide an estimate of the sample size needed to obtain a 95 % HDCI that excludes zero. The 95 % HDCI for Cohen’s d was established through non-parametric bootstrapping (Efron and Tibshirani, 1994). Specifically, we resampled the current subset of data 100 times and calculated Cohen’s d for the difference between variable x and variable y each time. We then calculated the mean and standard deviations of Cohen’s d across the 100 resampled values. The 95 % CI was determined by subtracting 1.96 times the standard deviation of the Cohen’s d values from the mean Cohen’s d value, and by adding 1.96 times the standard deviation of the Cohen’s d values to the mean Cohen’s d value.

**Fig. 1:**
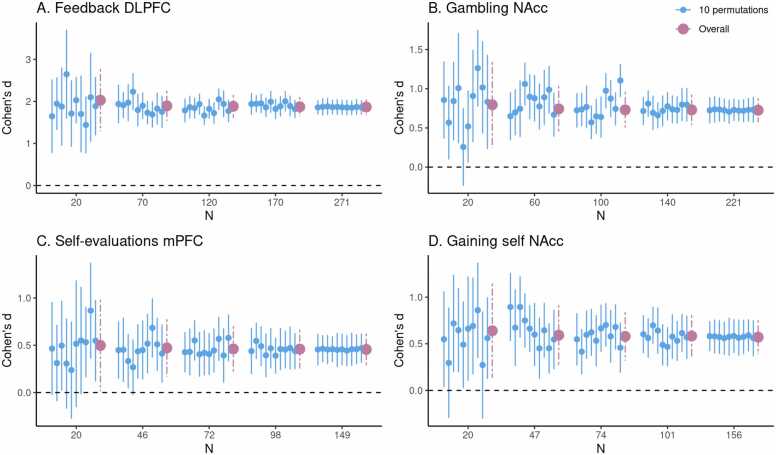
Estimates of task effects for five different sample sizes (starting with N=20, then 1/5th parts of the total dataset). For each sample size 10 randomly chosen HDCI’s out of the 1000 HDCI’s computed are displayed (in light blue). The average estimate with credible interval summarizing the 1000 HDCI’s for each sample size are plotted in reddish purple. DLPFC = dorsolateral prefrontal cortex; mPFC = medial prefrontal cortex; NAcc = nucleus accumbens.

**Fig. 2:**
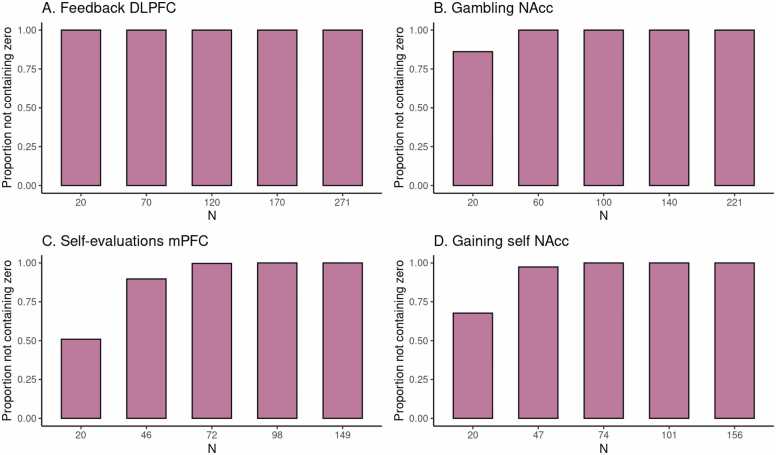
For each task, for five different sample sizes (starting with n=20, then 1/5th parts of the total dataset), the proportion of intervals not containing the value 0 is plotted in reddish purple.

To illustrate the current method, we can take a preview at Fig. 1 C in the Results section. The dataset used to construct HDCI’s for Cohen’s d consists of 149 participants from the self-evaluation versus control contrast in the medial prefrontal cortex during a self-evaluation task. In Fig. 1 C the first 20 participants in each of 1000 permuted data sets are used to compute the HDCI for Cohen’s d. Ten of these intervals are displayed in blue. As can be seen, of the ten (of 1000) HDCI’s displayed, four include the value zero. Consequently, fluctuations in the order in which participants are sampled determine whether the HDCI contains the value zero. This is summarized in Fig. 2 C which shows that the probability that one of the 1000 HDCI’s does not contain the value zero is about 0.6 for n=20. In Fig. 1 C, it is shown in the first interval displayed in purple that the average of the 1000 HDCI’s does not contain the value zero. Therefore, although the average interval does not contain the value zero, we can learn from the permutations that when using 20 participants it is still rather likely that the resulting interval will include the value zero. This becomes much less likely for the samples of 46 and 72 participants. With these sample sizes neither the individual HDCI’s nor the average interval contain the value zero (see Fig. 1 C). Additionally, the probability that an interval does not contain the value zero equals 100 % for 72 participants (see Fig. 2 C). Therefore, a sample size of 72 and higher will usually render intervals that do not contain the value zero.

An issue that sample size determination has in common with power analysis, is that the effect size in the existing data set will very likely differ from the effect size in the population that will be addressed in the study being designed. However, sample size determination does not suffer from the fact that effect sizes reported in the literature tend to be inflated (like power analysis) because effect sizes are straightforwardly computed from actual data. This also means that compared to power analyses, fewer assumptions about the data to be collected are needed. On the other hand, just like traditional power analyses, it is important to consider to what extent the existing data resemble the to be planned study. The determined sample size is therefore an estimate of the required sample size. This estimate is valuable because it allows researchers to plan their research project, can be reported in their research proposal or pre-registration, and it may lead to the conclusion that the sample size needed cannot be achieved with the resources that you have at your disposal. After planning, in the data collection phase, one can also estimate the HDCI at regular intervals with incoming data. Based on the outcome, additional data can be collected if needed or the researcher can decide that the precision of the HDCI is sufficient, and no more data collection is required. This will be especially useful in cases where resources are scarce, such as smaller studies conducted by individual research labs, or to limit participant burden.

In the next section Bayesian empirical sample size determination will be exemplified and discussed using different fMRI tasks from the BrainTime (Braams et al., 2014, Peters and Crone, 2017) and Self-Concept (Spaans et al., 2023, Cruijsen et al., 2023) data sets.

### 1.3 *neuroUp* R package

The code for Bayesian updating in this manuscript is implemented in the R language for statistical computing (R Core Team, 2023). We provide the *neuroUp* package at the following location: https://github.com/eduardklap/neuroUp and via the Comprehensive R Archive Network (CRAN): https://doi.org/10.32614/CRAN.package.neuroUp (Klapwijk, Hoijtink, and Jongerling, 2024). This package is built on several packages from the tidyverse (Wickham et al., 2019), most notably *ggplot2*. The package can be installed using the R command: install.packages("neuroUp"). All data used in the current manuscript are also available within the *neuroUp* package, in this way all figures in the manuscript can be reproduced using the R package. See Box 1 for a description of the usage of the package, and for a more elaborate introduction to *neuroUp* and its functions, please refer to https://eduardklap.github.io/neuroUp/articles/neuroUp.html. To cite package *neuroUp* in publications use Klapwijk, Hoijtink, and Jongerling (2024).

Box 1 **Data processing using *neuroUp***

The function estim_diff() helps to determine the sample size required to estimate differences in raw means and Cohen’s d’s for multiple sample sizes with a certain precision. Using the *neuroUp* package, we run the following code (with explanation below):

**Figure:**
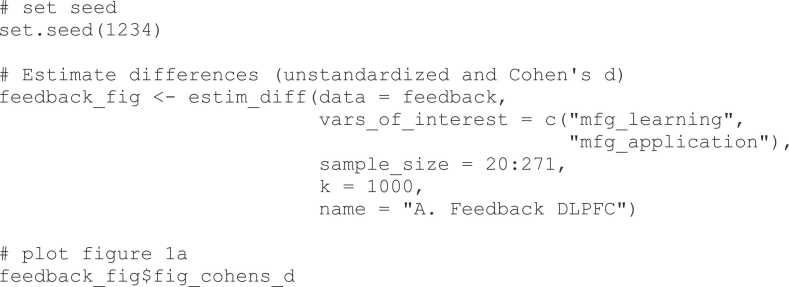

The first step is to set a seed value to make sure we get the same results when re-running the function (set.seed(1234)). Next, we store the outcomes of the estim_diff() function into a new object that we call feedback_fig. For the feedback task, we provide the feedback data set that comes with the *neuroUp* package as the data to be analyzed. As variables of interest (vars_of_interest) we provide a vector containing the names of the variables to be compared on their means (in our case, c("mfg_learning", "mfg_application")). Next, sample_size is the range of sample size to be used (20:271 in our example), and k is the number of permutations to be used for each sample size (1000) for the purpose of the current manuscript. Finally, name is an optional title of the dataset or variables to be displayed with the figures ("Feedback DLPFC" in our example). Once the estimations are ready (note: with 1000 permutations this may take more than 1 hour), in the final step the figure is plotted (feedback_fig$fig_cohens_d).

Subsequently, we run the same estim_diff() function for the Gambling, Self-Evaluations, and Vicarious Charity data sets that are internal to the *neuroUp* package. See the Supplementary notebooks for the code to create all figures and results reported below.

A reproducible version of this manuscript including associated code notebooks is available here: https://eduardklap.github.io/sample-size-fmri/. The code notebooks are also presented as supplementary data for this article. A CODECHECK (Nüst and Eglen, 2021) certificate is available confirming that the computations underlying this article could be independently executed: https://doi.org/10.5281/zenodo.13945051 (Röseler, 2024)*.*

## 2 Method and materials

We focus on sample size estimation for regions of interest from four different fMRI tasks in two different samples (see Table 1). We re-used data that has been processed with SPM8 or SPM12 and derived summary statistics for individual participants for regions and task conditions of interest (see fMRI processing).

### 2.1 Data and processing

#### 2.1.1 BrainTime study

The BrainTime study is an accelerated longitudinal research project of normative development. A total of 299 participants between ages 8 and 25 years took part in MRI scanning at the first time point. At the second time point approximately 2 years later, 254 participants were scanned (most dropout was due to braces, n=33). At time point 3, approximately 2 years after time point 2, 243 participants were scanned (dropout due to braces: n=11). The same Philips 3 T MRI scanner and settings were used for all time points. The following settings were used: TR = 2200ms, TE = 30ms, sequential acquisition, 38 slices, slice thickness = 2.75mm, field of view (FOV) = 220×220×114.68mm. A high-resolution 3D T1 anatomical scan was acquired (TR = 9.76ms, TE = 4.59ms, 140 slices, voxel size = 0.875×0.875×1.2mm, FOV = 224×177×168mm).

##### 2.1.1.1 Feedback task

For the feedback task (Peters et al., 2014) we included 271 participants at time point 1 from which region of interest data was available. We use the same data and processing as reported in Peters and Crone (2017). During the task, on each trial participants viewed three squares with a stimulus presented underneath. Participants were instructed to use performance feedback to determine which stimulus belonged to which square. After each choice, feedback was presented to the participant: a minus sign for negative feedback or a plus sign for positive feedback. After 12 trials, or when a criterion was reached (placing each of the three stimuli in the correct box at least two times), a new sequence with three new stimuli was presented. This criterion was used to strike a balance between the number of trials in the learning and the application phase. There were 15 sequences in total, resulting in a maximum of 15x12=180 trials per participant. For the current study, we focused on the learning > application contrast, which compares feedback early in the learning process with feedback for associations that were already learned (i.e. application phase). Individual trial-by-trial analyses were used to determine the learning and application phase (see Peters and Crone 2017 for details).

##### 2.1.1.2 Gambling task

For the Gambling task (Braams et al., 2014), we used data from 221 participants at time point 3 (we went for the largest sample size and this was the time point with the most data available for the contrasts of interest). We use the same data and processing as reported in Schreuders et al. (2018). In the Gambling task, participants could choose heads or tails and win money when the computer selected the chosen side of the coin or lose money when the opposite side was selected. Chances of winning were 50 %. To keep participants engaged, the number of coins that could be won or lost on varied across trials. Participants were explained that the coins won in the task would translate to real money, to be paid out at the end of the experiment. Gambling was performed in two different conditions: participants played 23 trials for themselves, and 22 trials for their best friend. For the current study, we focused on reward processing by analysing the winning for self > losing for self contrast.

#### 2.1.2 Self-Concept study

The Self-Concept study is a cohort-sequential longitudinal study in which 160 adolescents from 11 to 21 years old took part at the first time point (see https://osf.io/h7u46 for study overview). MRI scans were acquired on a Philips 3 T MRI scanner with the following settings: TR = 2200ms, TE = 30ms, sequential acquisition, 37 slices, slice thickness = 2.75mm, FOV = 220×220×111.65mm. A high-resolution 3D T1 anatomical scan was acquired (TR = 9.8ms, TE = 4.6ms, 140 slices, voxel size = 0.875×0.875×1.2mm, FOV = 224×178.5×168mm).

##### 2.1.2.1 Self-evaluations task

For the Self-evaluation task we used the same data and processing as reported in Cruijsen et al. (2023). In this task, participants read 60 short sentences describing positive or negative traits in the academic, physical, or prosocial domain (20 per domain; 10 with a positive valence and 10 with a negative valence). Here we focus on the direct self-evaluation condition, in which participants had to indicate to what extent the trait sentences applied to them on a scale of 1 (‘not at all’) to 4 (‘completely’). In the control condition, participants had to categorize 20 other trait sentences according to four categories: (1) school, (2) social, (3) appearance, or (4) I don’t know. For the current study, we focused on the direct self-evaluation versus control contrast. Eleven participants were excluded due to excessive motion during scanning (|3mm,n=8), not completing the scan (n=1), and a technical error (n=2), resulting in a total sample of N=149.

##### 2.1.2.2 Vicarious charity task

For the Vicarious Charity task, we used the same data and processing as reported in Spaans et al. (2023). In this task, participants could earn money for themselves and for a self-chosen charity they selected before scanning. On every trial, participants could choose one of two curtains to open. After their button press, the chosen curtain opened to show the division of the stake between themselves and the charity. The overall outcome was a division of either €4 or €2 between parties. Unknown to the participant, every outcome condition occurred 15 times during the task. Participants were informed beforehand that they received extra money based on the average of three random outcomes at the end of the research visit. Here we focus on the self-gain > both-no-gain contrast. This contrast compares the 30 trials in which participants gained the full €4 or €2 themselves (i.e., €0 for charity) against 15 baseline trials in which no money was gained in total (i.e., €0 for self and €0 for charity). Two participants were excluded from the analysis due to excessive movement during scanning (>3mm), one due to signal dropout in the SPM mask including the ventral striatum (this was assessed by visual inspection of all individual SPM masks), and one due to a technical error, resulting in a total of N=156.

#### 2.1.3 fMRI analysis

FMRI analyses for all four tasks were performed using SPM (Wellcome Department of Cognitive Neurology, London); SPM8 was used for the BrainTime data and SPM12 for the Self-Concept data. The following preprocessing steps were used: slice timing correction, realignment (motion correction), normalization, and smoothing (6 mm full-width at half maximum isotropic Gaussian kernel). T1 templates were based on the MNI305 stereotaxic space. All tasks have an event-related design and events were time-locked with 0 duration to the moment of feedback presentation. Six motion regressors were added to the model. All other trials (i.e., trials that did not result in learning in the Feedback task or too-late trials) were modeled as events of no interest. These events were used as covariates in a general linear model together with a set of cosine functions that high-pass filtered the data. The least-squares parameter estimates of height of the best-fitting canonical hemodynamic response function (HRF) for each condition were used in pair-wise contrasts. The contrast images were submitted to higher-level group analyses. Region of interest analyses were performed with the MarsBaR toolbox (Brett et al., 2002).

##### 2.1.3.1 Regions of interest

For the Feedback task, the atlas-based middle frontal gyrus was used as a region of interest (Harvard-Oxford cortical atlas; thresholded at 50 %; center-of-mass coordinates x=−4,y=22,z=43). Extracted parameter estimates for this region were obtained in tabular format from the authors of Peters and Crone (2017), with one value for the mean activation during learning and one value for the mean activation during application for all participants. For the Gambling task, the anatomical mask of the left nucleus accumbens was used as a region of interest (Harvard-Oxford subcortical atlas; thresholded at 40 %; 28 voxels included). Extracted parameter estimates for this region were obtained in tabular format from the authors of Schreuders et al. (2018), with one value for the mean activation during winning and one value for the mean activation during losing for all participants.

For the Self-evaluation task, the region of interest used was a mask of the left medial prefrontal cortex (x=−6,y=50,z=4) based on a meta-analysis by Denny et al. (2012). Extracted parameter estimates for this region were obtained in tabular format from the authors of Cruijsen et al. (2023), with one value for the mean activation during self-evaluation and one value for the mean activation during the control condition for all participants. For the Vicarious Charity task, the anatomical mask of the left nucleus accumbens was used as a region of interest (Harvard-Oxford subcortical atlas; thresholded at 40 %; center-of-mass coordinates x=−10,y=12,z=−7; 28 voxels included). Extracted parameter estimates for this region were obtained in tabular format from the authors of Spaans et al. (2023), with one value for the mean activation during gaining for self and one value for the mean activation during no-gain for self and charity for all participants.

## 3 Results

### 3.1 Task effects using Cohen’s d

The average HDCI was used to determine the optimal sample size for the four fMRI tasks used. For each task and brain region of interest, we estimated the sample size required to obtain an average HDCI for Cohen’s d not containing the value zero (Fig. 1 and Fig. 2; Table 2). Additionally, we also established whether it was also the case that a majority of the 1000 underlying HDCI’s did not contain the value zero.

**Table 2:** Mean estimates (with credible interval in brackets) of Cohen’s d for five different sample sizes (starting with n = 20, then 1/5th parts of the total dataset) of the 1000 HDCI’s. DLPFC = dorsolateral prefrontal cortex; mPFC = medial prefrontal cortex; NAcc = nucleus accumbens.

task	brain region	n = 20	n = 2/5	n = 3/5	n = 4/5	N = total
Feedback	DLPFC	2.03 (1.29, 2.76)	1.89 (1.54, 2.25), n = 70	1.88 (1.62, 2.15), n = 120	1.87 (1.65, 2.1), n = 170	1.87 (1.69, 2.04), *N* = 271
Gambling	NAcc	0.8 (0.25, 1.34)	0.74 (0.45, 1.03), n = 60	0.73 (0.51, 0.96), n = 100	0.73 (0.54, 0.92), n = 140	0.73 (0.58, 0.88), *N* = 221
Self-evaluations	mPFC	0.5 (0.02, 0.98)	0.47 (0.17, 0.77), n = 46	0.46 (0.22, 0.7), n = 72	0.46 (0.26, 0.66), n = 98	0.46 (0.29, 0.62), n = 149
Gaining self	NAcc	0.64 (0.14, 1.14)	0.59 (0.27, 0.91), n = 47	0.58 (0.32, 0.84), n = 74	0.58 (0.36, 0.8), n = 101	0.57 (0.39, 0.75), *N* = 156

In Fig. 1 A, an estimate of the required sample size for feedback learning processing in the DLPFC (middle frontal gyrus) is presented. Five groups of HDCI’s are presented for sample sizes of 20, 70, 120, 170, and 271 persons, in which 271 is the total group sample size of the existing data set. As can be seen, already with a sample of 20 participants neither of the 10 permuted HDCI’s displayed nor the average of the 1000 HDCI’s contain the value zero. In Fig. 2A it can be seen that the proportion of HDCI’s not containing the value zero is equal to 1.0. This is due to the huge effect size of Cohen’s d equal to about 1.9 (see Table 2). Therefore, already with a sample size of 20 participants, an effect bigger than zero will be detected for this task and brain region.

For the task effect of gambling (winning for self > losing for self contrast) in the NAcc, we can see the results in Fig. 1 B. Here, with a sample of 20 participants the average of the 1000 HDCI’s does not contain the value zero but some of the 10 HDCI’s displayed do. The proportion of HDCI’s not containing the value zero is bigger than 0.8 for 20 participants, but with 60 or more participants none of the 1000 HDCI’s contain zero anymore (Fig. 2B). This is related to the large effect size of Cohen’s d equal to about 0.7 (see Table 2). Thus, for this task and brain region, already with a sample size of 20 participants chances are high that an effect bigger than zero will be detected. Using sample sizes of 60 or more participants from this existing data set increases the chances of detection to 100 %.

In Fig. 1 C, results are plotted for the self-evaluation task in the mPFC. We see that at a sample size of 20 some of the 10 HDCI’s displayed still contain the value 0, but not the average of the 1000 HDCI’s. The proportion of HDCI’s not containing the value 0 is also below 1.0, around 0.5, for this task at N=20 (Fig. 2C). At the next step that is plotted for this task (n=46), we see that the proportion of HDCI’s not containing the value 0 is only a little below 1.0. Thus, for this task and brain region, at a sample size at 46 participants chances are high that an effect bigger than zero will be detected.

The results of gaining for self in the NAcc are plotted in Fig. 1 D. With a sample of 20 participants the average of the 1000 HDCI’s does not contain the value zero but some of the 10 HDCI’s displayed do. The proportion of HDCI’s not containing the value zero is around 0.75 (Fig. 2D), but at n=47 the proportion of HDCI’s not containing the value 0 is almost 1.0. Therefore, using 20 participants it is still likely that the resulting interval will include the value zero. This becomes much less likely for the samples of 47 and 74 persons. With these sample sizes neither the HDCI’s nor the average interval contain the value zero. Additionally, the probability that an interval does not contain the value zero almost reaches 100 %. Therefore, for this task and brain region, a sample size of 47 and higher will usually render intervals that do not contain the value zero.

### 3.2 Pearson correlations between task effects and age

**Fig. 3:**
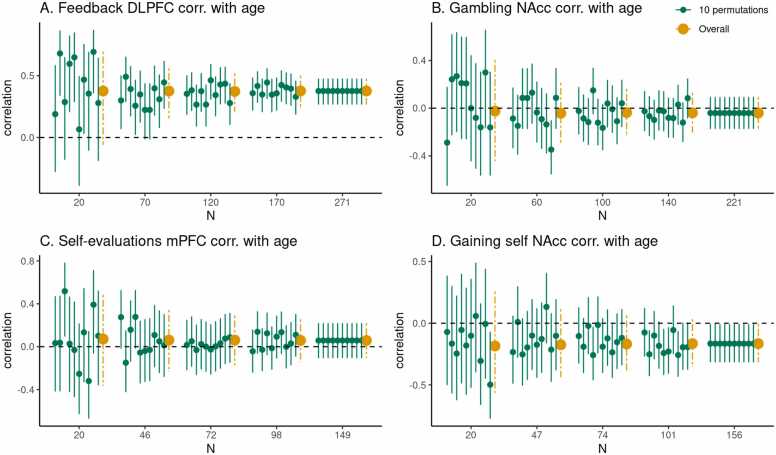
Estimates of Pearson’s correlation between age and the task effect for five different sample sizes (starting with N=20, then 1/5th parts of the total dataset). For each sample size 10 randomly chosen HDCI’s out of the 1000 HDCI’s computed are displayed (in green). The average estimate with credible interval summarizing the 1000 HDCI’s for each sample size are plotted in orange. DLPFC = dorsolateral prefrontal cortex; mPFC = medial prefrontal cortex; NAcc = nucleus accumbens. Age is modeled as linearly increasing or decreasing.

**Fig. 4:**
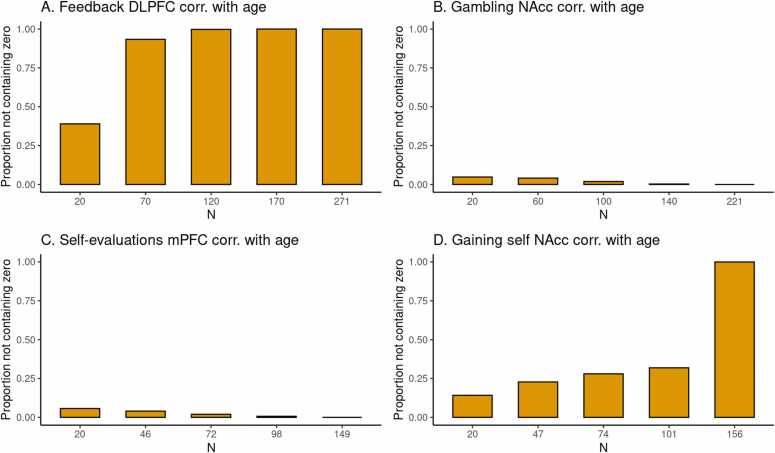
For each task, for five different sample sizes (starting with N=20, then 1/5th parts of the total dataset), the proportion of intervals not containing the value 0 is plotted inorange. Age is modeled as linearly increasing or decreasing.

For each task and brain region of interest we also estimated the sample size required to obtain an average HDCI for Pearson’s correlation between regional fMRI task responses and age not containing the value zero (Fig. 3 and Fig. 4; Table 3) for which a large proportion of the 1000 HDCI’s does not contain the value zero.

**Table 3:** Mean estimates (with credible interval in brackets) of Pearson’s correlation between age and the task effect for five different sample sizes (starting with N = 20, then 1/5th parts of the total dataset) of the 1000 HDCI’s. DLPFC = dorsolateral prefrontal cortex; mPFC = medial prefrontal cortex; NAcc = nucleus accumbens.

task	brain region	n = 20	n = 2/5	n = 3/5	n = 4/5	N = total
Feedback	DLPFC	0.38 (−0.06, 0.69)	0.38 (0.16, 0.56), *N* = 70	0.37 (0.21, 0.52), *N* = 120	0.38 (0.24, 0.5), *N* = 170	0.38 (0.27, 0.47), *N* = 271
Gambling	NAcc	−0.02 (−0.44, 0.4)	−0.04 (−0.29, 0.21), *N* = 60	−0.04 (−0.23, 0.16), *N* = 100	−0.04 (−0.2, 0.13), *N* = 140	−0.04 (−0.17, 0.09), *N* = 221
Self-evaluations	mPFC	0.07 (−0.36, 0.48)	0.06 (−0.23, 0.34), *N* = 46	0.06 (−0.17, 0.29), *N* = 72	0.06 (−0.14, 0.25), *N* = 98	0.06 (−0.1, 0.22), *N* = 149
Gaining self	NAcc	−0.18 (−0.56, 0.26)	−0.17 (−0.44, 0.11), *N* = 47	−0.17 (−0.38, 0.06), *N* = 74	−0.17 (−0.35, 0.03), *N* = 101	−0.17 (−0.31, −0.01), *N* = 156

In Fig. 3 A the results are shown for the Pearson’s correlation between age (children and adolescents aged 8–25 years) and feedback learning processing in the DLPFC. As can be seen, with a sample of 20 persons the average HDCI contains the value 0. Increasing the sample size to 70 results in most of the 10 HDCI’s displayed and the average of the 1000 HDCI’s not containing the value 0. We can also see that the proportion of HDCI’s not containing the value zero is less than 0.5 for 20 participants but closer to 1.0 for 70 participants (Fig. 4 A). Therefore, for a reliable estimate of the correlation between feedback processing in the DLPFC and age, sample sizes bigger than 20–70 are needed.

For the correlation between age and gambling in the NAcc, we can see the results in Fig. 3 B. Here, for all plotted sample sizes both the average of the HDCI’s and all of the 10 HDCI’s displayed do contain the value zero. The proportion of HDCI’s not containing the value zero is below 0.1 for 20 participants, but with more participants almost all the 1000 HDCI’s contain the value zero (Fig. 4 B). This is related to the very small correlation equal to about −0.04 (see Table 3). Thus, for this task and brain region, it is unlikely that an effect larger than zero will be detected for a correlation with age, even with the total sample size of 221 participants.

In Fig. 3 C, results are plotted for the correlation between age and self-evaluation processing in the mPFC. We see that for all plotted sample sizes both the average of the 1000 HDCI’s and most of the 10 HDCI’s displayed do contain the value zero. In Fig. 4 C, we also see that the proportion of HDCI’s not containing the value 0 is well below 1.0 for all sample sizes shown. Thus, for this task and brain region, with all available sample sizes the chances are high there will be no effect detected that is larger than zero. The average Pearson correlation is also small with a value around 0.06 (Table 3). The results for the correlation between age and gaining for self in the NAcc are plotted in Fig. 3D. We see that up to 101 participants both the average of the 1000 HDCI’s and most of the 10 HDCI’s displayed do contain the value zero. The proportion of HDCI’s not containing the value zero increases with bigger sample sizes (Fig. 4 A) but is still below 0.5 for 101 participants. Therefore, with 101 participants it is still likely that the resulting interval will include the value zero. Therefore, for this task and brain region, with all available sample sizes the chances are high there will be no effect detected that is larger than zero. The average Pearson correlation is also relatively small with a value around −0.17 (Table 3). If correlations of about −0.17 are considered relevant, the conclusion might be that one could strive for a sample larger than 156.

## 4 Discussion

In this paper we tested how existing data can be used to approximately determine the sample size that is required for a new study that is being planned. The approach presented has been illustrated using four existing fMRI studies where Cohen’s d is the effect size of interest and four studies where a Pearson correlation for linear age is of interest. Additionally, it has been elaborated that Bayesian updating can be used to deal with the fact that the required sample size can only approximately be determined.

Our results show that calculating and plotting HDCI’s can be helpful in determining at what sample sizes task effects become more stable. We illustrated with four examples how researchers can get an indication of the required sample size if they plan to execute a study that uses feedback learning, reward-processing (two examples), or self-processing. For all effects under study, the width of the HDCI became smaller with larger sample sizes, showing that the precision of the estimation increased with more data. At what sample size estimations seemed to stabilize for general contrasts (e.g., feedback processing versus rule application; reward versus loss) differed per task and region of interest. For example, the effects in the middle frontal gyrus during feedback processing were the strongest of the effects studied here. There, even for sample sizes of 20 participants, task effects clearly differed from zero for the middle frontal gyrus. But even for such a strong effect, at higher sample sizes the results became more stable (i.e., less variability in subsamples of the total dataset). For the other three tasks, the HDCI’s still contained the value zero with 20 participants but at higher sample sizes of 40–60 participants chances are very high that an effect larger than zero will be detected.

The results are different for the Pearson correlation between linear age and task effects, as can be seen in Fig. 3 and Fig. 4. Here again the results of the middle frontal gyrus during feedback processing were the strongest. But even for such a strong effect, the sample size needed to estimate the correlation with age is much higher than the sample size needed to find a stable task effect. For the Pearson correlation, only at a sample size of N=70 or more the lower bound of the credible is above zero, suggesting that one can be confident that there is a positive Pearson correlation.

For the correlation between linear age (between 12 and 28 years) and reward-processing in the NAcc, the results were dependent on the specific task context. In a gambling task with the contrast reward versus loss, even with a sample size of 221 the average HDCI contains the value zero when testing for linear age effects. We found that the average Pearson correlation is −.04 for this effect, which is so small that it is likely irrelevant. In contrast, for rewards in a vicarious reward context with the contrast reward versus no reward, probability of a positive Pearson correlation with negative age increased with larger sample sizes and the average CI did just not contain zero using the full sample size of 156 ($M=-0.17;95\%\mathrm{CI}\left[-0.31,-0.01\right]$). Note that we only tested for linear age effects, whereas prior studies reported that reward processing may be best described by non-linear effects (Braams et al. 2014); which should be tested in future extensions of the approach illustrated here. The results described for the linear age effects are in line with simulation studies suggesting that typically in psychology relatively large sample sizes of N=150 to N=250 (Schönbrodt and Perugini, 2013) are needed to obtain a study that is sufficiently powered to evaluate a Pearson correlation with age or psychological variables.

There are different ways in which the methods described here could be put to practice. When planning to use an existing task for follow-up or replication research, existing data can be used to provide an educated guess of the sample size that is needed in the new study (given that the new samples resemble the existing sample). When doing so, it is important to keep in mind that there will always be differences in study execution that might influence the required sample size (e.g., differences in task presentation, scan parameters, number of trials) (Bennett and Miller, 2013, Chen et al., 2022). In those cases, it is up to the researcher to decide to what extent the existing studies resemble the study they are planning. This means that instead of providing a definitive answer about the number of participants needed, our approach serves as a tool that helps estimating the sample size. In this way new task-related fMRI studies will likely be properly powered, even if these are small-scale studies from individual labs. Even in the current era of large-scale consortium studies, there remains the need to conduct studies with more modest sample sizes. Such studies can help to balance between methodological robustness of well-established experiments and experimental design novelty. These data sets could complement large, public data sets with more idiosyncratic tasks and specific samples. Furthermore, sample size determination using existing data can be combined with Bayesian updating when collecting new data for a new research project. The following steps can be used:

1. Execute Bayesian empirical sample size determination. This will render an estimate of the sample size needed for an adequately powered study.
2. Execute the planned study with the sample size determined in the previous step.
3. Compute the effect size of interest (e.g., Cohen’s d or Pearson’s correlation) and the corresponding HDCI.
4. Evaluate the HDCI:
  a. If the HDCI does not contain the value zero, your study is finished.
  b. If the HDCI does contain the value zero, there are two options:
    i. If the estimate of the effect size is close to zero, the effect size is small. Adding more data (updating) may (or may not) render an HDCI that does not contain the value but will very likely still render an effect size that is so small that it is irrelevant.
    ii. If the estimated effect size is relevant in the context of the study being executed, it may very well be (but there is no certainty) that collecting additional data (updating) will render a HDCI that does not contain the value zero. If you have the resources, it may be worthwhile to collect additional data and return to Step 3). If you do not have the resources, your study is finished.

In this paper the focus was on Cohen’s d and the Pearson correlation using cross-sectional data, however, the approach presented can straightforwardly be generalized to other statistical models and effect size measures. A relatively simple example is quadratic regression with either Spearman’s correlation or the multiple correlation as the effect size measure, but generalizations to more complex statistical models such as multilevel models applied to longitudinal data are conceivable. One specifically interesting avenue would be a combination of Bayesian updating and power contours (Baker et al., 2021) to consider the combined effect of the number of participants tested and number of trials per participant (Chen et al., 2022). Arguably, in more complex statistical models our approach may succeed where classical power analysis may very well fail. The reason for the latter is that in power analysis “the effect size” has to be specified. In, for example, structural equation models, this implies that factor loadings, regression coefficients and (co)variances have to be specified. Doing this such that the specification represents a certain “effect size” is at least difficult and maybe even impossible. In our approach effect sizes do not have to be specified but are straightforwardly estimated using existing data. Therefore, in an era of open science in which data sets are increasingly available for re-use (also for sample size determination) there is much potential for the approach presented in this paper (see Box 2 for more example use cases).

Box 2 **Use cases**

**Example case 1: reward learning task main effect**

In this box, we illustrate the use of this method in a dataset that is not from our own lab as an additional use case. We provide an example of a researcher who is planning a new study in which the researcher is interested in the main effect of reward versus no-reward conditions in the caudate nucleus during a reward learning task. For this example, we used existing data from Calabro et al. (2023) for 232 participants in total (including additional developmental data), with permission from the authors. In the first step we run the estimations, which suggest that we should aim for a sample size of at least 104 participants, because in this case all estimations are above zero. As can be seen in the figures below, estimations become more robust at n = 146 and n = 232 but the advantage of more data above n = 146 is relatively small.

**Figure:**
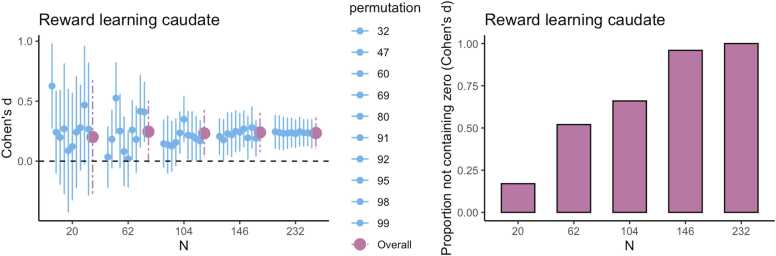

In the following step, the researcher starts the new study with the sample size determined using existing data with a comparable paradigm. This means the researcher can administer a (new) reward learning task in a sample of 104 participants.

The researcher computes the HDCI’s for the new data. In case most of the permutations do not contain the value zero, data collection can be finished. However, it is also possible to collect additional data in case the new results are less robust; in this case the HDCI’s can be computed again with more data. Taken together, the method presented here provides a valid estimate of the required sample size for starting a new study.

**Example case 2: reward learning task age effect**

In case a researcher is interested in correlations with age or other predictor variables (e.g. IQ or SES), the procedure should be performed again with the predictor variable included. Using the data from Calabro et al. (2023), we provide an example of a researcher who is planning a new study in which they are interested in the relationship between age and the parametric association between caudate nucleus activation and reward prediction error (for which we have one value per participant). In the first step we run the estimations, which suggest that in case we are interested in linear age effects, we should aim for a sample size of at least 146 participants. As can be seen in the Figures below, at n = 146 the HDCI’s contain zero for a large proportion of the permutations. This suggests that for more robust correlational effects, it is recommended to include at least 146 participants in a new study.

**Figure:**
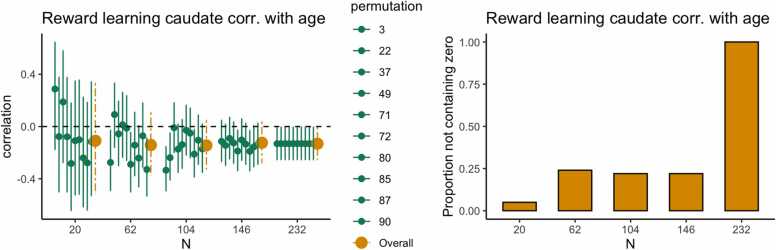

### 4.1 Practical recommendations

When calculating HDCI’s using existing data, we should keep in mind that the determined sample size is only an indication of the sample size required for the new study. In the new study the unknown effect size (Cohen’s d or Pearson’s correlation) may be smaller or larger than in the existing study and therefore a somewhat smaller or larger sample size may be required. This can be accommodated using Bayesian updating in the data collection phase with the determined sample size as the point of departure. The HDCI can be estimated at regular intervals with incoming data, which can help to determine whether additional data are needed or not. It is also important to consider how one can determine the resemblance of a “to be planned study” to the existing study. Although this is a subjective evaluation, it is probably wise to be mindful of different factors that can influence the strength of the effect, such as task domain, number of trials, scan parameters, region of interest, and modality (e.g., visual versus verbal) (Bennett and Miller, 2013, Elliott et al., 2020, Herting et al., 2018). In case of correlational studies, the range of the independent variable such as age should also be considered. One can undersample from the existing data at hand, in case the age range of interest of the planned study falls within that range. But when the age range of interest is wider than the range of the data at hand, extrapolation of the estimates to the new sample should be done with caution.

Carefully considering the differences in study design between existing and planned studies is also important for cases where the ultimate consequence of the sample size estimation might be that one should not execute the planned study. For example, if a study resembles study B in Fig. 3, it is to be expected that the effect size for linear correlations with age (if one expects a linear and not a non-linear effect) is so small that either it is irrelevant, or unrealistic large sample sizes are required to obtain an average HDCI that does not contain zero. But even in this situation, it depends on the context whether something is a meaningful small effect or a negligible small effect (see for further discussions Dick et al. 2021 and Funder and Ozer 2019). One might still decide to continue the study when there is evidence that the small effect at hand is meaningful, for example because cumulatively it can lead to larger effects in the longer run. If the effect is yet deemed irrelevant, one could choose not to collect new data, or one could alternatively take a bet to pilot a novel version of a similar task that might lead to a stronger effect. Bayesian updating can then be used to estimate the HDCI’s again after a small number of participants. If the average effect is still close to zero one might conclude it is probably better to use a different paradigm or to focus on other aspects of the study.

In conclusion, in this paper we demonstrate that the current HDCI approach may be a helpful tool in planning a study. The quantification of the required sample is most valid if the existing study characteristics and study samples are comparable to the planned study. Yet, in most cases (apart from direct replications), new research is carried out to discover something new by implementing a variation of a task in a new sample or setting. Our approach can then also be used to monitor the precision of the estimates and compare it with the existing dataset (by plotting the estimates at certain predetermined sample size intervals). That is also another way in which the approach can be used for the interesting cases in which a totally new task is used. Together, we aimed to present a user-friendly open source tool to determine sample sizes based on existing data for paradigms that were validated in previous research, or in the process of data collection in case the study tests scientific questions using novel paradigms.

## CRediT authorship contribution statement

**Eveline Crone:** Writing – review & editing, Supervision, Methodology, Investigation, Funding acquisition, Conceptualization. **Herbert Hoijtink:** Writing – review & editing, Visualization, Supervision, Software, Methodology, Conceptualization. **Joran Jongerling:** Writing – review & editing, Visualization, Validation, Software, Methodology. **Eduard Klapwijk:** Writing – review & editing, Writing – original draft, Visualization, Software, Formal analysis, Data curation.

## Declaration of Competing Interest

The authors declare that they have no known competing financial interests or personal relationships that could have appeared to influence the work reported in this paper.

## Data Availability

The processed data required to reproduce the above findings are available to download from https://doi.org/10.5281/zenodo.11526169 (as part of the *neuroUp* R package).

## Footnotes

1 In statistical terms: the HDCI contains 95 % of the marginal posterior density of the parameter which is a function of the information in the data and prior knowledge with respect to the parameter.
