# Supplementary material for: Sample size estimation for task-related functional MRI studies using Bayesian updating
Source: Dev Cogn Neurosci. 2024 Dec 17;71:101489. doi: 10.1016/j.dcn.2024.101489 (PMC11732471; doi:10.1016/j.dcn.2024.101489)
Supplement: Supplementary file 1 — Supplementary material [file mmc1.zip › figures-cohens_d_V3.html]

Code for Figures 1-2 based on Cohen’s d


# Code for Figures 1-2 based on Cohen’s d

Author

Eduard Klapwijk

Load libraries to produce figures:

```
# load libraries
library(dplyr)
library(patchwork)
library(ggplot2)
library(ggpubr)
# install neuroUp package from CRAN
# install.packages("neuroUp")
# load neuroUp
library(neuroUp)
```

Set seed and create Figure 1a for the Feedback task DLPFC ROI:

```
# set seed
set.seed(1234)

# Estimate differences (unstandardized and Cohen's d)
feedback_fig <- estim_diff(data = feedback,
                           vars_of_interest = c("mfg_learning",
                                                "mfg_application"),
                           sample_size = 20:271, 
                           k = 1000, 
                           name = "A. Feedback DLPFC")

# plot figure 1a (and remove legend using ggplot2)
feedback_fig$fig_cohens_d + theme(legend.position = "none")
```

Figure 1

Plot Figure 2a for the Feedback task DLPFC ROI:

```
# plot figure 2a
feedback_fig$fig_d_nozero
```

Figure 2

Set seed and create Figure 1b for the Gambling task NAcc ROI:

```
# set seed
set.seed(1234)

# Estimate differences (unstandardized and Cohen's d)
gambling_fig <- estim_diff(data = gambling, 
                           vars_of_interest = c("lnacc_self_win", 
                                                "lnacc_self_loss"), 
                           sample_size = 20:221, 
                           k = 1000, 
                           name = "B. Gambling NAcc")
# plot figure 1b (and remove legend using ggplot2)
gambling_fig$fig_cohens_d + theme(legend.position = "none")
```

Figure 3

Plot Figure 2b for the Gambling task NAcc ROI:

```
# plot figure 2b
gambling_fig$fig_d_nozero
```

Figure 4

Set seed and create Figure 1c for the Self-evaluations task mPFC ROI:

```
# set seed
set.seed(1234)

# Estimate differences (unstandardized and Cohen's d)
selfeval_fig <- estim_diff(data = self_eval, 
                           vars_of_interest = c("mpfc_self", 
                                                "mpfc_control"),
                           sample_size = 20:149, 
                           k = 1000, 
                           name = "C. Self-evaluations mPFC")

# plot figure 1c (and remove legend using ggplot2)
selfeval_fig$fig_cohens_d + theme(legend.position = "none")
```

Figure 5

Plot Figure 2c for the Self-evaluations task mPFC ROI:

```
# plot figure 2c
selfeval_fig$fig_d_nozero
```

Figure 6

Set seed and create Figure 1d for the Gaining for self task NAcc ROI:

```
# set seed
set.seed(1234)

# Estimate differences (unstandardized and Cohen's d)
vicar_char_fig <- estim_diff(data = vicar_char, 
                             vars_of_interest = c("nacc_selfgain", 
                                                  "nacc_bothnogain"),
                             sample_size = 20:156, 
                             k = 1000, 
                             name = "D. Gaining self NAcc")

# plot figure 1d (and remove legend using ggplot2)
vicar_char_fig$fig_cohens_d + theme(legend.position = "none")
```

Figure 7

Plot Figure 2d for the Gaining for self task NAcc ROI:

```
# plot figure 2d
vicar_char_fig$fig_d_nozero
```

Figure 8

Plot a mock figure with similar aesthetics to create an overall ggplot legend.

The only purpose of this code chunk is to make a simple overall legend to show that in light blue a subset of the individual permutations is shown and in purple the overall values. The original legends with permutation numbers will not be shown in the overall figure to create a cleaner look. The approach taken here was to use a simple mock ggplot figure and then use the `ggpubr` package to isolate the legend and display the legend together with the 4 actual plots.

```
# make simple mock data to create overall legend
legend_data <- tibble(legend = c("10 permutations","Overall"), N = 1:2, scores = c(1.6, 1.8),
                      lower = c(.4, .6), upper = c(2.8, 3))
# factorize legend and N
legend_data$legend <- factor(legend_data$legend)
legend_data$N <- as.factor(legend_data$N)

# plot data to produce legend
figure_legend <- ggplot2::ggplot(data = legend_data, 
                               ggplot2::aes(x = .data$N, 
                                            y = .data$scores,
                                            colour = .data$legend,
                                            size = .data$legend) ) +
  ggplot2::theme_classic() +
  ggplot2::geom_point(position = ggplot2::position_dodge(.8),
                      ggplot2::aes(x = .data$N, 
                                   y = .data$scores,
                                   colour = .data$legend,
                                   size = .data$legend)) +
  ggplot2::scale_size_manual(values = c(2, 4))  +
  ggplot2::geom_errorbar(ggplot2::aes(ymin = .data$lower, ymax = .data$upper),
                         linewidth = .5, 
                         position = ggplot2::position_dodge(.1)) +
  ggplot2::scale_linetype_manual(values = c(1, 6)) +
  ggplot2::scale_color_manual(values = c("#56B4E9","#CC79A7") ) +
  theme(legend.title=element_blank())

# use ggpubr get_legend to plot legend only
leg <- ggpubr::get_legend(figure_legend)
simple_legend <- ggpubr::as_ggplot(leg)

# show the simple overall legend
simple_legend
```

Figure 9

Plot Figure 1 (4 data sets combined):

```
# plot figure 1 using patchwork library
feedback_fig$fig_cohens_d + theme(legend.position = "none") + 
  gambling_fig$fig_cohens_d + theme(legend.position = "none") + 
  selfeval_fig$fig_cohens_d + theme(legend.position = "none") + 
  vicar_char_fig$fig_cohens_d + theme(legend.position = "none") +
  inset_element(simple_legend, 
                left = 1.7, bottom = 3.8, right = 0, top = 0, 
                on_top = T, align_to = 'full')
```

Figure 10: Estimates of task effects for five different sample sizes (starting with \(N=20\), then 1/5th parts of the total dataset). For each sample size 10 randomly chosen HDCI’s out of the 1000 HDCI’s computed are displayed (in light blue). The average estimate with credible interval summarizing the 1000 HDCI’s for each sample size are plotted in reddish purple. DLPFC = dorsolateral prefrontal cortex; mPFC = medial prefrontal cortex; NAcc = nucleus accumbens.

Plot Figure 2 (4 data sets combined):

```
# plot figure 2 using patchwork library
feedback_fig$fig_d_nozero + ylab(label = "Proportion not containing zero") +
  gambling_fig$fig_d_nozero + ylab(label = "Proportion not containing zero") + 
  selfeval_fig$fig_d_nozero + ylab(label = "Proportion not containing zero") +
  vicar_char_fig$fig_d_nozero + ylab(label = "Proportion not containing zero")
```

Figure 11: For each task, for five different sample sizes (starting with \(n=20\), then 1/5th parts of the total dataset), the proportion of intervals not containing the value 0 is plotted in reddish purple.

Extract numbers to make table 2:

```
# first extract tibble from results (select mean only)
feedback_sum <- as_tibble(feedback_fig$tbl_select) %>%
  dplyr::filter(permutation == "Overall")
gambling_sum <- as_tibble(gambling_fig$tbl_select) %>%
  dplyr::filter(permutation == "Overall")
selfeval_sum <- as_tibble(selfeval_fig$tbl_select) %>%
  dplyr::filter(permutation == "Overall")
gaining_sum <- as_tibble(vicar_char_fig$tbl_select) %>%
  dplyr::filter(permutation == "Overall")
```

Use numbers to produce a table in text:

| task | brain region | n = 20 | n = 2/5 | n = 3/5 | n = 4/5 | N = total |
| --- | --- | --- | --- | --- | --- | --- |
| Feedback | DLPFC | **2.03** (1.29, 2.76) | **1.89** (1.54, 2.25), *n* = 70 | **1.88** (1.62, 2.15), *n* = 120 | **1.87** (1.65, 2.1), *n* = 170 | **1.87** (1.69, 2.04), *N* = 271 |
| Gambling | NAcc | **0.8** (0.25, 1.34) | **0.74** (0.45, 1.03), *n* = 60 | **0.73** (0.51, 0.96), *n* = 100 | **0.73** (0.54, 0.92), *n* = 140 | **0.73** (0.58, 0.88), *N* = 221 |
| Self-evaluations | mPFC | **0.5** (0.02, 0.98) | **0.47** (0.17, 0.77), *n* = 46 | **0.46** (0.22, 0.7), *n* = 72 | **0.46** (0.26, 0.66), *n* = 98 | **0.46** (0.29, 0.62), *N* = 149 |
| Gaining self | NAcc | **0.64** (0.14, 1.14) | **0.59** (0.27, 0.91), *n* = 47 | **0.58** (0.32, 0.84), *n* = 74 | **0.58** (0.36, 0.8), *n* = 101 | **0.57** (0.39, 0.75), *N* = 156 |
