# Supplementary material for: Sample size estimation for task-related functional MRI studies using Bayesian updating
Source: Dev Cogn Neurosci. 2024 Dec 17;71:101489. doi: 10.1016/j.dcn.2024.101489 (PMC11732471; doi:10.1016/j.dcn.2024.101489)
Supplement: Supplementary file 2 — Supplementary material [file mmc2.zip › figures-correlations_V3.html]

Code for Figures 3-4 based on Pearson’s correlations


# Code for Figures 3-4 based on Pearson’s correlations

Author

Eduard Klapwijk

Load libraries to produce figures:

```
# load libraries
library(dplyr)
library(patchwork)
library(ggplot2)
library(ggpubr)
library(neuroUp)
```

Set seed and create Figure 3a for the Feedback task DLPFC ROI:

```
# set seed
set.seed(1234)

# calculate difference mfg_learning vs. mfg_application
data_feedback <- feedback
data_feedback$dif <- data_feedback$mfg_learning - data_feedback$mfg_application

# Estimate correlations
feedback_fig <- estim_corr(data = data_feedback,
                           vars_of_interest = c("dif", "age"),
                           sample_size = 20:271, 
                           k = 1000, 
                           name = "A. Feedback DLPFC corr. with age")
# plot figure 3a
feedback_fig$fig_corr
```

Figure 1

Plot Figure 4a for the Feedback task DLPFC ROI:

```
# plot figure 4a
feedback_fig$fig_corr_nozero
```

Figure 2

Set seed and create Figure 3b for the Gambling task NAcc ROI:

```
# set seed
set.seed(1234)

# Estimate correlations
gambling_fig <- estim_corr(data = gambling, 
                           vars_of_interest = c("lnacc_self_winvsloss", 
                                                "age"), 
                           sample_size = 20:221, 
                           k = 1000, 
                           name = "B. Gambling NAcc corr. with age")

# plot figure 3b
gambling_fig$fig_corr
```

Figure 3

Plot Figure 4b for the Gambling task NAcc ROI:

```
# plot figure 4b
gambling_fig$fig_corr_nozero
```

Figure 4

Set seed and create Figure 3c for the Self-evaluations task mPFC ROI:

```
# set seed
set.seed(1234)

# calculate difference mpfc_self - mpfc_control
data_self <- self_eval
data_self$dif <- data_self$mpfc_self - data_self$mpfc_control

# Estimate correlations
selfeval_fig <- estim_corr(data = data_self,
                           vars_of_interest = c("dif",
                                                "age"),
                           sample_size = 20:149, 
                           k = 1000, 
                           name = "C. Self-evaluations mPFC corr. with age")

# plot figure 3c
selfeval_fig$fig_corr
```

Figure 5

Plot Figure 4c for the Self-evaluations task mPFC ROI:

```
# plot figure 4c
selfeval_fig$fig_corr_nozero
```

Figure 6

Set seed and create Figure 3D for the Gaining for self task NAcc ROI:

```
# set seed
set.seed(1234)

# calculate difference nacc_selfgain vs. nacc_bothnogain
data_vicar <- vicar_char
data_vicar$dif <- data_vicar$nacc_selfgain - data_vicar$nacc_bothnogain

# Estimate correlations
vicar_char_fig <- estim_corr(data = data_vicar, 
                             vars_of_interest = c("dif", 
                                                  "age"),
                             sample_size = 20:156, 
                             k = 1000, 
                             name = "D. Gaining self NAcc corr. with age")

# plot figure 3d
vicar_char_fig$fig_corr
```

Figure 7

Plot Figure 4d for the Gaining for self task NAcc ROI:

```
# plot figure 4d
vicar_char_fig$fig_corr_nozero
```

Figure 8

Plot a mock figure with similar aesthetics to create an overall ggplot legend.

The only purpose of this code chunk is to make a simple overall legend to show that in light blue a subset of the individual permutations is shown and in purple the overall values. The original legends with permutation numbers will not be shown in the overall figure to create a cleaner look. The approach taken here was to use a simple mock ggplot figure and then use the `ggpubr` package to isolate the legend and display the legend together with the 4 actual plots.

```
# make simple mock data to create overall legend
legend_data <- tibble(legend = c("10 permutations","Overall"), N = 1:2, scores = c(1.6, 1.8),
                      lower = c(.4, .6), upper = c(2.8, 3))
# factorize legend and N
legend_data$legend <- factor(legend_data$legend)
legend_data$N <- as.factor(legend_data$N)

# plot data to produce legend
figure_legend <- ggplot2::ggplot(data = legend_data, 
                               ggplot2::aes(x = .data$N, 
                                            y = .data$scores,
                                            colour = .data$legend,
                                            size = .data$legend) ) +
  ggplot2::theme_classic() +
  ggplot2::geom_point(position = ggplot2::position_dodge(.8),
                      ggplot2::aes(x = .data$N, 
                                   y = .data$scores,
                                   colour = .data$legend,
                                   size = .data$legend)) +
  ggplot2::scale_size_manual(values = c(2, 4))  +
  ggplot2::geom_errorbar(ggplot2::aes(ymin = .data$lower, ymax = .data$upper),
                         linewidth = .5, 
                         position = ggplot2::position_dodge(.1)) +
  ggplot2::scale_linetype_manual(values = c(1, 6)) +
  ggplot2::scale_color_manual(values = c("#009E73", "#E69F00") ) +
  theme(legend.title=element_blank())

# use ggpubr get_legend to plot legend only
leg <- ggpubr::get_legend(figure_legend)
simple_legend <- ggpubr::as_ggplot(leg)

# show the simple overall legend
simple_legend
```

Figure 9

Plot Figure 3 (4 data sets combined):

```
# plot figure 3 using patchwork library
feedback_fig$fig_corr + theme(legend.position = "none") +
  gambling_fig$fig_corr + theme(legend.position = "none") + 
  selfeval_fig$fig_corr + theme(legend.position = "none") +
  vicar_char_fig$fig_corr + theme(legend.position = "none") +
  inset_element(simple_legend, 
                left = 1.7, bottom = 3.8, right = 0, top = 0, 
                on_top = T, align_to = 'full')
```

Figure 10: Estimates of Pearson’s correlation between age and the task effect for five different sample sizes (starting with \(N=20\), then 1/5th parts of the total dataset). For each sample size 10 randomly chosen HDCI’s out of the 1000 HDCI’s computed are displayed (in green, permutation numbers used are displayed to the right of each subfigure). The average estimate with credible interval summarizing the 1000 HDCI’s for each sample size are plotted in orange. DLPFC = dorsolateral prefrontal cortex; mPFC = medial prefrontal cortex; NAcc = nucleus accumbens. Age is modeled as linearly increasing or decreasing.

Plot Figure 4 (4 data sets combined):

```
# plot figure 4 using patchwork library
feedback_fig$fig_corr_nozero + gambling_fig$fig_corr_nozero + selfeval_fig$fig_corr_nozero + vicar_char_fig$fig_corr_nozero
```

Figure 11: For each task, for five different sample sizes (starting with \(N=20\), then 1/5th parts of the total dataset), the proportion of intervals not containing the value 0 is plotted in orange. Age is modeled as linearly increasing or decreasing.

Extract numbers to produce table 3:

```
# first extract tibble from results (select mean only)
feedback_sum <- as_tibble(feedback_fig$tbl_select) %>%
  dplyr::filter(permutation == "Overall")
gambling_sum <- as_tibble(gambling_fig$tbl_select) %>%
  dplyr::filter(permutation == "Overall")
selfeval_sum <- as_tibble(selfeval_fig$tbl_select) %>%
  dplyr::filter(permutation == "Overall")
gaining_sum <- as_tibble(vicar_char_fig$tbl_select) %>%
  dplyr::filter(permutation == "Overall")
```

Use numbers to produce a table in text:

| task | brain region | n = 20 | n = 2/5 | n = 3/5 | n = 4/5 | N = total |
| --- | --- | --- | --- | --- | --- | --- |
| Feedback | DLPFC | **0.38** (-0.06, 0.69) | **0.38** (0.16, 0.56), *n =* 70 | **0.37** (0.21, 0.52), *n =* 120 | **0.38** (0.24, 0.5), *n =* 170 | **0.38** (0.27, 0.47), *N* = 271 |
| Gambling | NAcc | **-0.02** (-0.44, 0.4) | **-0.04** (-0.29, 0.21), *n =* 60 | **-0.04** (-0.23, 0.16), *n =* 100 | **-0.04** (-0.2, 0.13), *n =* 140 | **-0.04** (-0.17, 0.09), *N =* 221 |
| Self-evaluations | mPFC | **0.07** (-0.36, 0.48) | **0.06** (-0.23, 0.34), *n =* 46 | **0.06** (-0.17, 0.29), *n =* 72 | **0.06** (-0.14, 0.25), *n =* 98 | **0.06** (-0.1, 0.22), *N* = 149 |
| Gaining self | NAcc | **-0.18** (-0.56, 0.26) | **-0.17** (-0.44, 0.11), *n =* 47 | **-0.17** (-0.38, 0.06), *n =* 74 | **-0.17** (-0.35, 0.03), *n =* 101 | **-0.17** (-0.31, -0.01), *N* = 156 |
